# Sepsis due to kidney injury caused by a toothpick: a case report and literature review

**DOI:** 10.1186/s12879-022-07058-2

**Published:** 2022-02-02

**Authors:** Qi Qi, Lingxin Chen, Guoxian Kou

**Affiliations:** The Infection Department of Mianyang Central Hospital, Mianyang, 621000 China

**Keywords:** Foreign body, Septicemia, Toothpick, Kidney injury, Case report

## Abstract

**Background:**

Toothpicks are common foreign bodies which may injure surrounding organs leading to a series of atypical symptoms. We present a rare clinical case that septicemia caused by a toothpick penetrated into the right kidney.

**Case presentation:**

We describe a 51-year-old patient who presented with right-sided backache and hematuresis for 2 days. Blood culture persistently grew *Streptococcus gordonii.* Ultrasound of the patient’s urinary tract revealed a strong striated echo in the middle of the right kidney. Complete abdominal computed tomography revealed a duodenal foreign body penetrating into the right kidney. The toothpick was removed under endoscopy and hemostasis was given. Antibiotic treatment was upgraded. The patient was recovered and discharged from his stay on the fifteenth day.

**Conclusions:**

Early identification of the etiology of sepsis can effectively alleviate patient’s distress and reduce hospital stay. Clinicians should identify the source of sepsis through a medical history and examination.

## Background

Toothpicks are a common foreign body in the digestive tract. Ingestion of a toothpick can lead to local or systemic abnormalities, such as perforation of the digestive tract and damage to surrounding organs. Its clinical manifestations vary from no clinical symptoms (spontaneous discharge of the foreign body in the digestive tract) to abdominal pain, gastrointestinal bleeding, dysphagia, fever, sepsis, and even death [1, 2]. Gastrointestinal foreign bodies may also cause rare renal injury, with atypical manifestations of hematuria and backache. At present, the main clinical methods to identify foreign bodies in the digestive tract are ultrasound examination, digestive tract endoscopy, laparoscopy, and open surgery. Although less common, in cases of kidney injury of unknown cause, the possibility of alimentary tract foreign bodies should be considered.

## Case presentation

A 51-year-old patient was admitted to the emergency department of our hospital with chief complaints of right-sided backache and hematuresis for 2 days, without fever or any other discomfort. At the emergency department, his laboratory results showed that white blood cell count and blood and urine neutrophils were abnormally elevated. Abdominal computed tomography (CT) showed peripheral exudation around the kidneys and the right ureter, which was obvious on the right side. The patient was transferred to the infection department with suspected urinary tract infection.

The patient had no special previous medical history. He had a history of alcohol consumption for more than 20 years. He denied any relevant family history. On physical examination, the patient was febrile, with a temperature of 38.9 °C. He had a pulse rate of 110 bpm, a respiration rate of 21 breaths/min, and a blood pressure of 131/77 mmHg. A cardiopulmonary examination showed no abnormal results, and the patient exhibited abdominal, renal region, and upper ureteral tenderness.

**Fig. 1:**
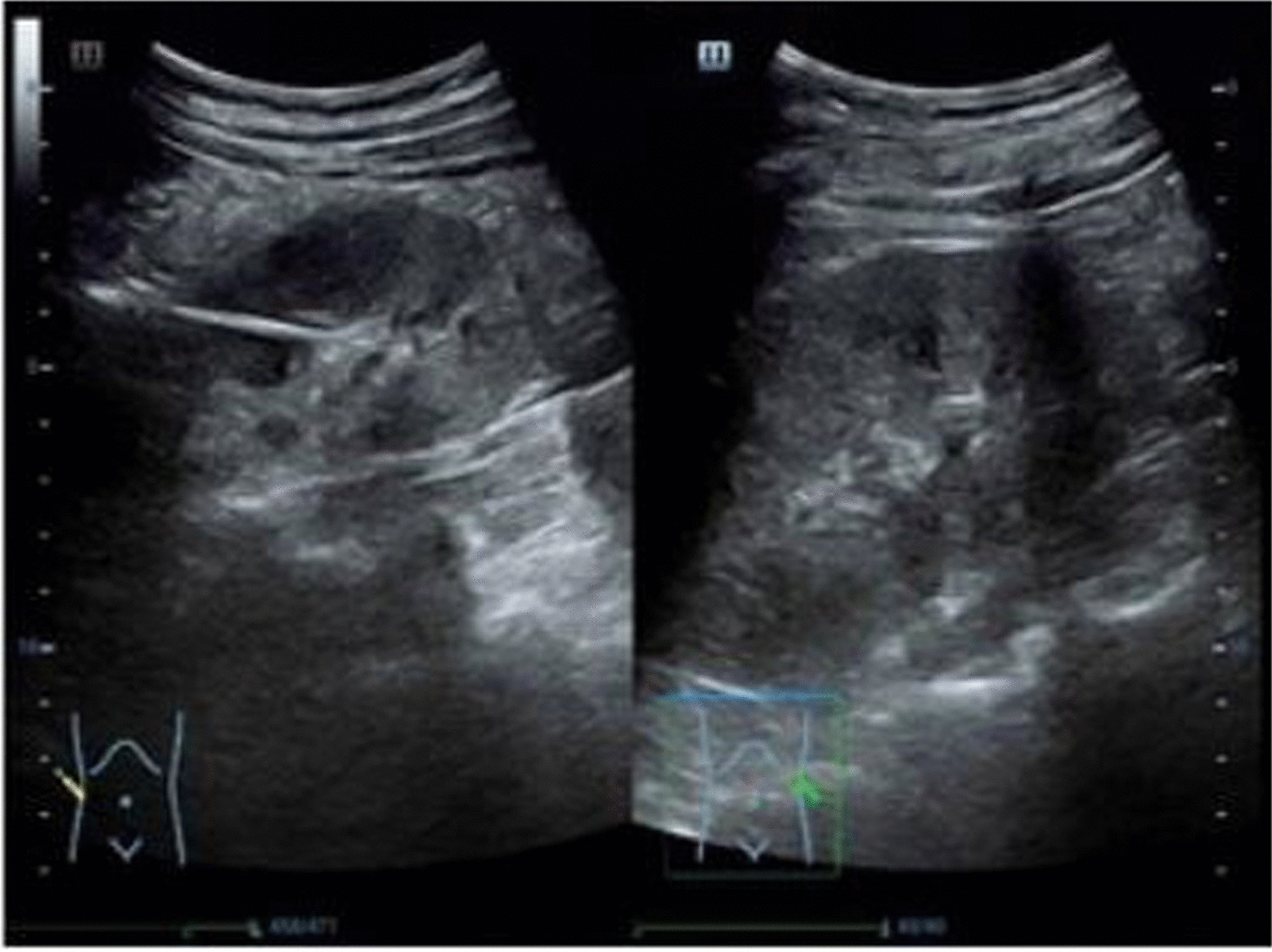
Strong stripe echo in the middle part of the right kidney was identified on ultrasound of the urinary system

**Fig. 2:**
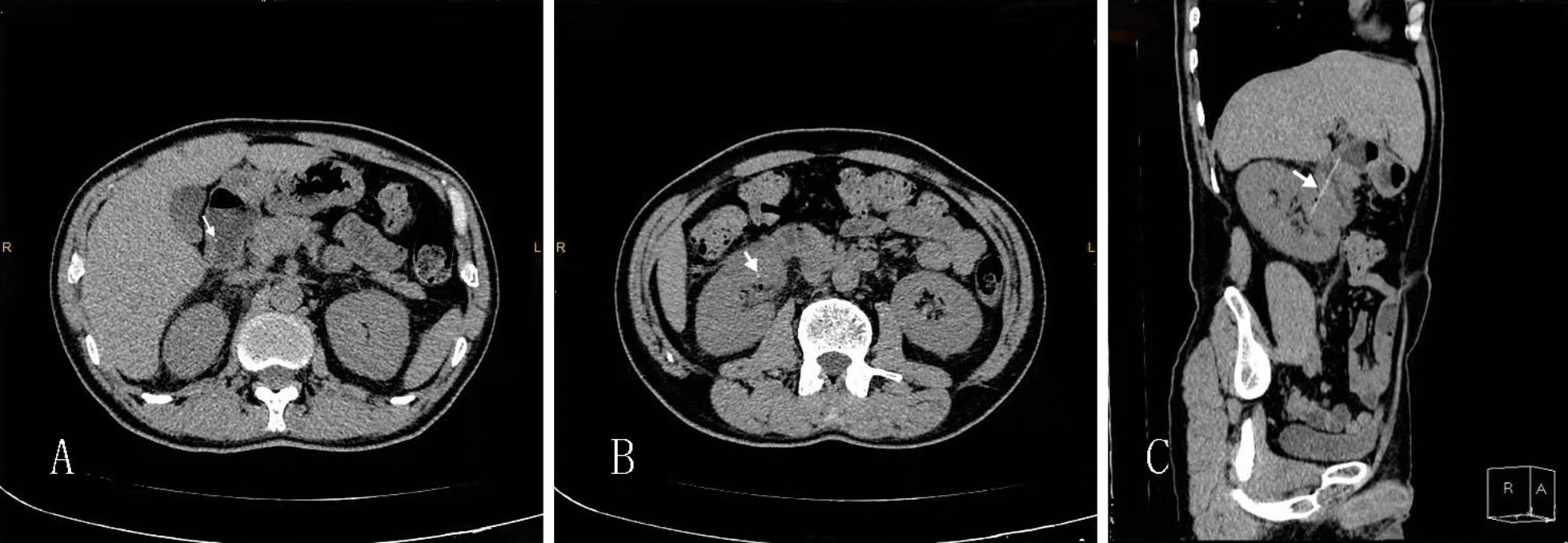
The two ends of toothpick are respectively showed as high density dots in duodenum (**A**: arrow) and the right kidney (**B**: arrow). The toothpick shown as linear high density is penetrated into the right kidney in the CT after 3D reconstruction (**C**: arrow). The “R” and “L” in pictures A and B represent the right and left respectively. In picture C, “R” and “A” represent the right side and ventral side respectively

Laboratory examination results were as follows: C-reactive protein, 122.6 μmol/L; procalcitonin, 0.438 μg/L; and *Streptococcus gordonii* was detected by blood culture. Ultrasound of the urinary system identified strong stripe echo in the middle part of the right kidney (Fig. 1). Considering the presence of a foreign body, we repeatedly requested the imaging department to re-review the abdominal CT scan. It was found that the duodenal foreign body had penetrated into the right kidney (Fig. 2).

**Fig. 3:**
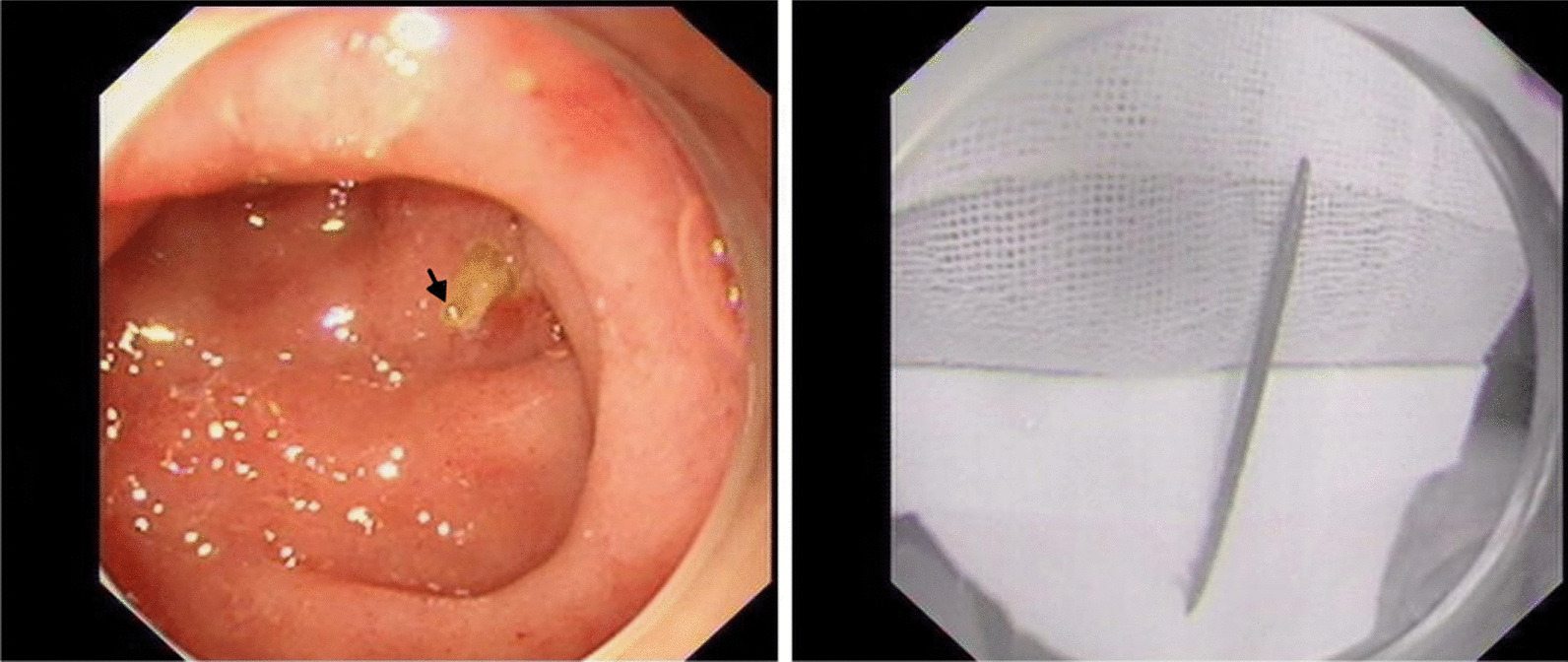
A toothpick in the duodenum was identified by endoscopy (arrow)

The final diagnoses were septicemia; a foreign body in the duodenum, which penetrated into the right kidney; and right kidney infection. We removed a 5-cm toothpick under guidance of digestive endoscopy (Fig. 3). The patient was instructed to fast and was treated with continued antibiotics and fluid nutrition support. The patient was asked about his medical history again and recalled that he might have accidentally ingested a toothpick when drinking alcohol 1 month prior. Considering that the patient had sepsis, we upgraded the antibiotic regimen from piperacillin/tazobactam to meropenem.

After 3 days, the patient underwent a second gastroscopy, which prompted chronic atrophic gastritis and duodenitis with dotted hemorrhage. White blood cells, neutrophils, and procalcitonin returned to normal. After 10 days of treatment, the patient resumed a normal diet. He did not present with fever, hematuria, or lumbago. He felt better and was discharged from the hospital on the 15th day. At the time of writing, the patient was undergoing regular follow up at the outpatient department.

## Discussion

Digestive tract foreign bodies are often observed in clinical practice, especially in children, the elderly, and patients with mental illness [3]. Digestive tract foreign bodies in adults are mostly related to alcohol consumption, which is more common in males. The patient in this case recalled that he might have swallowed a toothpick by mistake one month ago, but most patients cannot confirm or recall a history of accidental foreign body ingestion. Most digestive tract foreign bodies are passed out without causing any clinical symptoms. However, some foreign bodies, especially sharp ones, such as toothpicks and fish bones, can lead to gastrointestinal perforation, which in turn can lead to peritonitis, liver abscess, appendicitis, and sepsis, amongst other conditions [4, 5].

In this case, the patient did not recall a history of foreign body ingestion at admission. The patient’s blood culture was positive. The results of abdominal CT suggested that the only infectious focus that could be responsible for sepsis was perirenal urinary tract infection. However, according to our clinical experience of the different physiological structures between males and females, we recognized that the incidence of urinary tract infection in males is much lower compared with females. Thus, we deemed it necessary to further search for the infected focus. Therefore, our patient underwent urinary ultrasound, and the results indicated a foreign body in the renal area. Subsequently, we re-reviewed abdominal CT and identified a strip foreign body from the duodenum to the kidney. We then successfully removed the toothpick under gastroscopy. We searched PubMed for articles on perforation of the digestive tract and sepsis caused by toothpicks, as shown in additional file 1. In previous case reports, unidentified foreign bodies, which caused the exact infectious focus that led to infection in the present case, led to repeated hospitalization of patients with sepsis [6–10]. Therefore, it is vital for practitioners to actively search for the infectious focus in patients with fever and sepsis of unknown cause.

Pathogens that cause sepsis in this type of disease include fungi, gram-negative bacteria, gram-positive bacteria, and a mixture of multiple pathogens [8, 10–13]. Antibiotic regimens are usually based on carbapenems [9, 14–18]. Although blood culture suggested gram-positive bacterial infection (*Streptococcus gordonii*), we empirically chose meropenem, which is more sensitive to gram-negative bacteria for anti-infective therapy. This is because intestinal infections are dominated by gram-negative bacteria. During subsequent treatment, the patient’s body temperature returned to normal, and considering the effectiveness of antibiotic treatment, we did not change his antibiotic regimen.

According to previous literature reports, the most common gastrointestinal perforation sites after ingestion of a toothpick are the duodenum, small intestine, colon, and stomach, respectively [19]. We report a rare case of kidney injury caused by a duodenal foreign body. Only one previous case of renal injury was presented with hematuria, and an exploratory laparotomy was performed [20]. At present, the main treatment methods for such cases are gastroscopy, laparoscopy, and laparotomy. Early and timely detection of etiology can effectively reduce the course of disease, the pain experienced by patients, and examination costs.

In conclusion, septicemia and kidney injury caused by a duodenal foreign body are difficult to diagnose because of non-specific symptoms and a common unawareness of ingestive history. For clinicians, it is important to identify the etiology when the effect of antibiotic therapy is poor. To do this, clinicians should have a good knowledge of imaging.

## Data Availability

Not applicable.

## Abbreviation

CT: Computed tomography
